# Anterior Chamber Flare as a Non-Invasive Assessment of Intraocular Immune Status and Ocular Complications in Proliferative Diabetic Retinopathy

**DOI:** 10.3390/ijms25179158

**Published:** 2024-08-23

**Authors:** Tomohito Sato, Yuki Takenaka, Yoshiaki Nishio, Masataka Ito, Masaru Takeuchi

**Affiliations:** 1 Department of Ophthalmology, National Defense Medical College, Tokorozawa 359-8513, Saitama, Japan; dr21043@ndmc.ac.jp (T.S.); mii.52yy@gmail.com (Y.T.); cln349@ndmc.ac.jp (Y.N.); 2 Department of Developmental Anatomy and Regenerative Biology, National Defense Medical College, Tokorozawa 359-8513, Saitama, Japan; masataka@ndmc.ac.jp

**Keywords:** anterior chamber flare, aqueous humor, correlation, cytokine, diabetic macular edema, interleukin-8, proliferative diabetic retinopathy, receiver operating characteristic, vitreous fluid

## Abstract

Proliferative diabetic retinopathy (PDR) is a vision-threatening complication of diabetes mellitus (DM). Anterior chamber (AC) flare and intraocular cytokines are potent biomarkers reflecting the intraocular immune status in PDR. This study aimed to elucidate the complex interrelationship between AC flare and intraocular cytokines in PDR eyes. A retrospective observational study was conducted on 19 PDR eyes of 19 patients with type 2 DM, and on 19 eyes of 19 patients with idiopathic macular hole or epiretinal membrane as controls. AC flare was measured before pars plana vitrectomy (PPV). Aqueous humor (AH) and vitreous fluid (VF) samples were collected at the time of PPV, and the quantities of 27 cytokines in both intraocular fluids were analyzed. In the PDR and control groups, Spearman’s rank correlation analysis revealed a positive correlation between AC flare and IL-8 level in both AH and VF. Additionally, IL-8 levels in AH correlated positively with IL-8 levels in VF. In the PDR group, receiver operating characteristic curve analysis identified IL-8 level in AH as a significant predictor for both diabetic macular edema (DME) and vitreous hemorrhage (VH) complications. The cut-off values of IL-8 were established at ≥26.6 pg/mL for DME and ≥7.96 pg/mL for VH. Given the positive correlation between AC flare and AH IL-8 level, the present findings suggest that AC flare value may potentially be a non-invasive biomarker for predicting DME.

## 1. Introduction

Diabetic retinopathy (DR) is a severe microvascular complication of diabetes mellitus (DM) and a potentially vision-threatening condition affecting patients aged 20 to 75 years [1]. In Japan, the estimated annual incidence of DR in patients with DM is approximately 4% [2]. The global prevalence of DR among DM patients is around 35.4% [3]. Proliferative DR (PDR) is an advanced stage of DR characterized by vitreous hemorrhage (VH) and tractional retinal detachment (TRD) due to proliferative membrane traction [4]. Low-grade inflammation is known to be a driver of the disease process, and previous studies have implicated various systemic [5] and intraocular factors in the aqueous humor (AH) and vitreous fluid (VF) [6,7] in the pathogenesis and progression of DR.

Anterior chamber (AC) flare measured by laser flare photometry [8] has been used as a convenient and non-invasive quantitative biomarker of protein levels in the AC. Laboratory studies have shown a strong correlation between laser flare photometry measurements and protein concentrations in serially diluted protein solutions derived from plasma or AH samples [9,10]. AC flare has further been investigated as a potential inflammatory index for various ocular diseases including cataract [10], uveitis [11], retinitis pigmentosa [12,13], age-related macular degeneration (AMD) [14], DR [15,16], retinal vein occlusion [17] and proliferative vitreoretinopathy [18].

Cytokines are small, non-structural proteins secreted by diverse immune and non-immune cells, playing a pivotal role in inflammatory responses and cell proliferation regulation [19]. Cytokine levels in the AH and VF have emerged as potent biomarkers for elucidating intraocular immune status in PDR [4,20]. Previous studies have reported that inflammatory cytokines including interleukin (IL)-6, interferon gamma-induced protein 10 (IP-10), monocyte chemotactic protein 1 (MCP-1) and vascular endothelial growth factor (VEGF)-A are elevated in the VF of PDR patients compared to controls with idiopathic macular hole (MH) or idiopathic epiretinal membrane (ERM) [4,21]. Although analyzing cytokines in intraocular fluids provides valuable insights into PDR pathology, sampling of intraocular fluid presents a challenge due to the invasive nature of the procedure.

To the best of our knowledge, this study is the first comprehensive investigation of simultaneous measurements of AC flare and multiple intraocular cytokines in both the AC and VF of PDR eyes. We aimed to achieve two primary objectives: (1) to evaluate the correlation between AC flare and intraocular cytokines, and (2) to identify potential biomarkers for PDR complications, which can be derived from AC flare. This approach holds promise for establishing a non-invasive method for predicting intraocular cytokine levels, which could have significant implications for the diagnosis and monitoring of PDR complications.

## 2. Results

### 2.1. Demographic and Clinical Characteristics

The demographic and clinical characteristics of the PDR and control groups are summarized in Table 1. The mean age ± standard deviation was 61.5 ± 10.4 years (range 45–80 years) in the PDR group, and 66.1 ± 9.42 years (range 48–79 years) in the control group. The male-to-female ratio was 13/6 in the PDR group, and 8/11 in the control group. The logarithm of the minimum angle of resolution (logMAR) visual acuity (VA) was significantly higher in the PDR group than in the control group. No significant differences were observed in age, gender, laterality, intraocular pressure, and central retinal thickness (CRT) between the two groups.

### 2.2. Anterior Chamber Flare Intensity and Intraocular Cytokine Levels

Table 2 summarizes the profiles of AC flare and AH cytokines in PDR and control groups. The PDR group exhibited significantly higher AC flare intensity and AH levels of IL-8, eotaxin, IP-10, and macrophage inflammatory protein (MIP)-1α compared to the control group. The AH cytokines of IL-1 receptor antagonist (ra), IL-6, IL-7, IL-8, eotaxin, interferon-gamma (IFN-γ), IP-10, MCP-1, MIP-1α and MIP-1β had detection rates exceeding 20% in both groups. Additionally, the detection rates of IL-4, IL-9, regulated on activation, normal T-cell expressed and secreted (RANTES), and VEGF-A in AH were higher than 20% only in the PDR group.

Table 3 presents the VF cytokine profiles in the PDR and control groups. Similar to the AH cytokine findings, the PDR group showed significantly elevated levels of numerous VF cytokines compared to the control group. These cytokines comprised IL-6, IL-8, eotaxin, IFN-γ, IP-10, MCP-1, MIP-1α, MIP-1β, RANTES, and tumor necrosis factor alpha (TNFα).

### 2.3. Correlation between Anterior Chamber Flare and Aqueous Humor Cytokines

**Figure 1:**
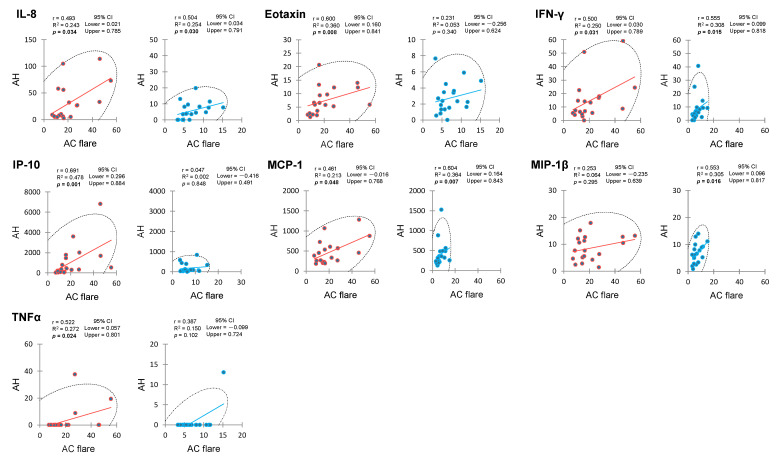
Significant correlation between anterior chamber flare and aqueous humor cytokines in eyes with proliferative diabetic retinopathy and controls. PDR group eyes in red on the left and control group eyes in blue on the right. The scales on both axes are symmetrical for each group to ensure that the slope of the graph is independent of the axis scale. Each graph includes 95% confidence intervals and confidence ellipses shown in black dotted lines. AC flare intensity (horizontal axis) is expressed in photon counts per millisecond (ph/ms). Cytokine levels (vertical axis) are expressed in pg/mL. AC—anterior chamber, AH—aqueous humor, CI—confidence interval, r—Spearman’s rank correlation coefficient, R^2^—coefficient of determination.

Table 4 presents the matrices of *p* values obtained from Spearman’s rank correlation analyses for AC flare intensity and AH cytokine levels in the PDR and control groups. The matrices illustrate the strength and direction of the correlation between AC flare and each of the cytokines in the two groups. In the PDR group, AC flare exhibited significant positive correlation with IL-8, eotaxin, IFN-γ, IP-10, MCP-1, and TNFα in AH (Figure 1). Additionally, all AH cytokines except IL-1ra demonstrated positive correlation with one or more of the other cytokines. The control group presented a different correlation pattern. AC flare showed significant positive correlation with IL-8, IFN-γ, MCP-1, and MIP-1β in AH (Figure 1). Similar to the PDR group, all AH cytokines except IL-1ra and IL-9 correlated with one or more of the other cytokines.

### 2.4. Aqueous Humor Cytokines with Causal Relationship to Anterior Chamber Flare

To investigate whether AH cytokine levels contribute to AC flare intensity, multiple regression analysis [22] was performed to explore the causal relationship between the two variables (Table 5). In the PDR group, IP-10 and MCP-1 were found to be independently associated with AC flare, consistent with the positive correlation in Spearman correlation analysis as shown in Table 4. These findings support the hypothesis that AC flare may be evaluated as an alternative biomarker of intraocular inflammatory cytokines in PDR eyes.

The control group presented a different pattern. The analysis identified IL-7 and MIP-1β in AH as significant factors for AC flare. Note that only MIP-1β correlated positively with AC flare in Spearman correlation analysis (Table 4).

### 2.5. Correlation between Anterior Chamber Flare and Vitreous Fluid Cytokines

**Figure 2:**
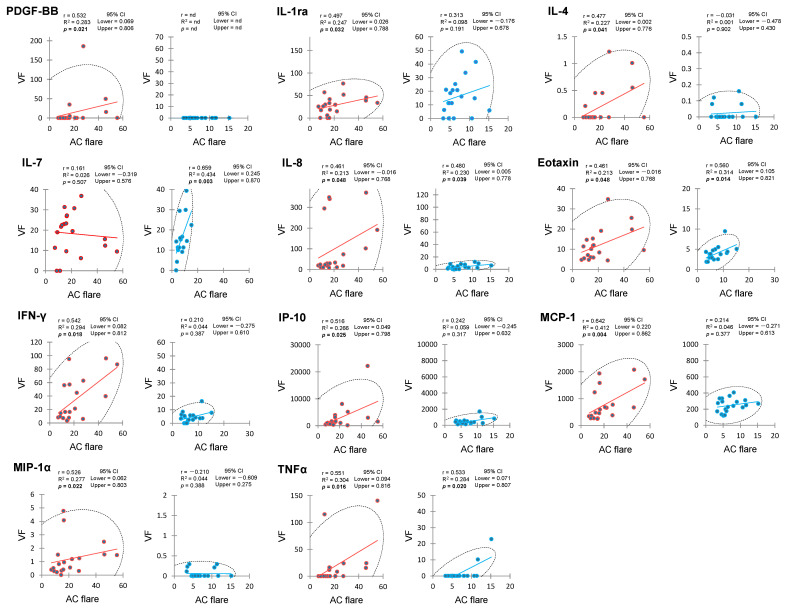
Significant correlation between anterior chamber flare and vitreous fluid cytokines in eyes with proliferative diabetic retinopathy and controls. VF—vitreous fluid.

Table 6 presents the matrices of the *p* values obtained from Spearman’s rank correlation analysis for AC flare and VF cytokines in the PDR and control groups. In the PDR group, AC flare exhibited significant positive correlation with a broader range of VF cytokines compared to the control group (Figure 2). These cytokines included platelet-derived growth factor-BB (PDGF-BB), IL-1ra, IL-4, IL-8, eotaxin, IFN-γ, IP-10, MCP-1, MIP-1α, and TNFα. Furthermore, all VF cytokines showed positive correlation with multiple other cytokines. The control group showed a more limited pattern of correlation. AC flare only correlated positively with IL-7, IL-8, eotaxin, and TNFα in VF. Interestingly, all VF cytokines except IL-13 demonstrated positive correlation with one or more of the other cytokines.

### 2.6. Correlation between Aqueous Humor and Vitreous Fluid Cytokines

**Figure 3:**
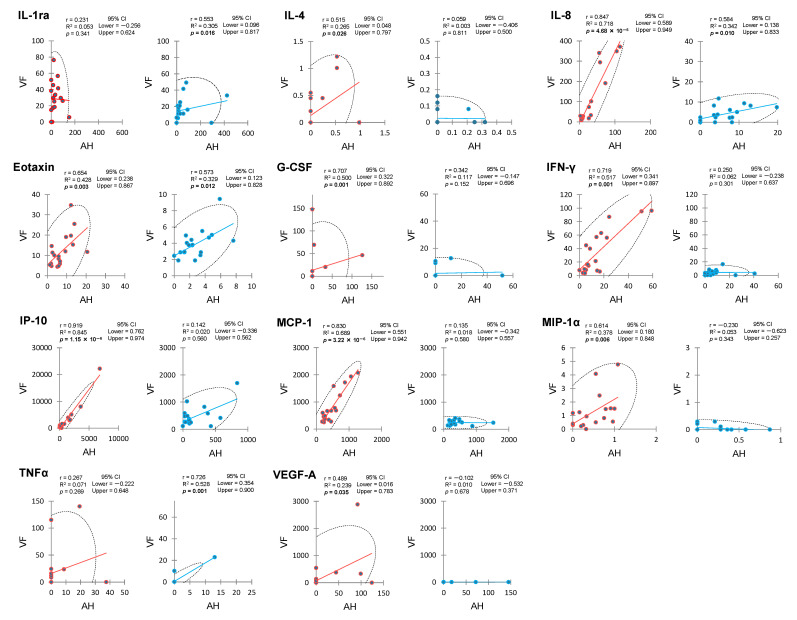
Significant correlation between aqueous humor and vitreous fluid cytokines in eyes with proliferative diabetic retinopathy and controls.

Figure 3 illustrates significant positive correlation between AH and VF cytokine levels in the PDR or control group. These cytokines comprised IL-4, IL-8, eotaxin, granulocyte colony-stimulating factor (G-CSF), IFN-γ, IP-10, MCP-1, MIP-1α, and VEGF-A. Conversely, no significant correlation between AH and VF levels was observed in the remaining cytokines of IL-1ra, IL-5, IL-6, IL-7, IL-9, IL-13, IL-17A, MIP-1β, RANTES, and TNFα (Table 7).

Figure 3 and Table 7 present significant positive correlation between AH and VF cytokine levels for IL-1ra, IL-8, eotaxin, and TNFα in the control group. No correlation between AH and VF levels was found for the remaining cytokines of IL-4, IL-6, IL-7, G-CSF, IFN-γ, IP-10, MCP-1, MIP-1α, MIP-1β, and VEGF-A.

In both PDR and control groups, intraocular IL-8 exhibited a consistent pattern of positive correlation between AC flare and AH IL-8 level, between AC flare and VF IL-8 level, and between AH and VF IL-8 levels. Additionally, in the PDR group, eotaxin, IFN-γ, IP-10, and MCP-1 showed positive correlation between AC flare, AH level and VF level, as IL-8.

### 2.7. Predictive Biomarkers for Complications of Diabetic Macular Edema, Traction Retinal Detachment, and Vitreous Hemorrhage

**Figure 4:**
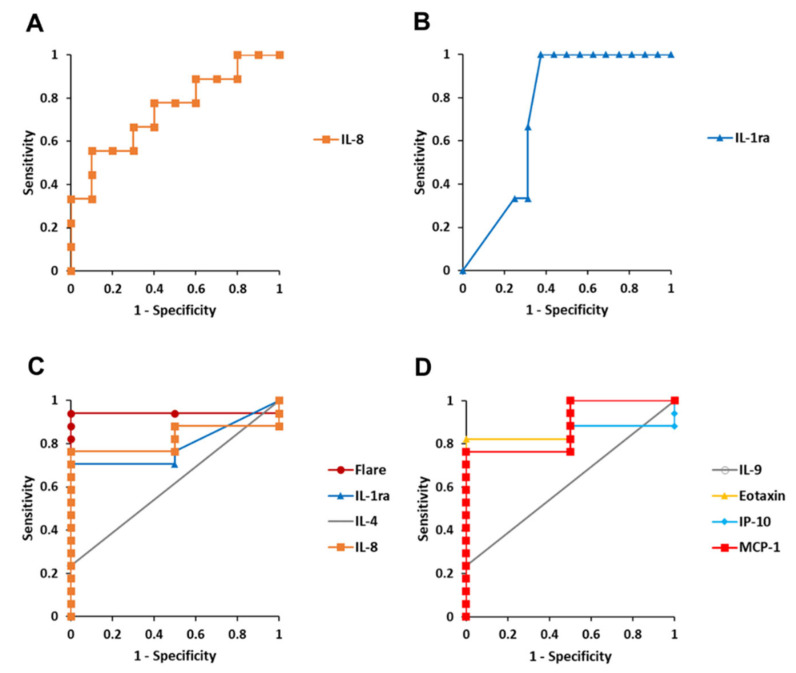
Predictive biomarkers of occurrence of diabetic macular edema (DME), traction retinal detachment (TRD), and vitreous hemorrhage (VH) in eyes with proliferative diabetic retinopathy. Receiver operating characteristic curves show that anterior chamber flare and aqueous humor levels of IL-1ra, IL-4, IL-8, IL-9, eotaxin, IP-10 and MCP-1 are significant predictive biomarkers for the risk of (**A**) DME, (**B**) TRD, and (**C**,**D**) VH. Cut-off value is defined by the point closest to the upper left-hand corner of the graph.

Among the measured parameters, AC flare and AH cytokines with detection rates exceeding 20% (Table 2) were selected for further analysis, considering the feasibility, reliability and accuracy of measurement. These factors were evaluated as potential predictive biomarkers for complications of DME, TRD, and VH using receiver operating characteristic (ROC) curve analysis. Figure 4 illustrates the ROC curves of significant predictors, and detailed results for all parameters analyzed are provided in Table 8. The AH level of IL-8 emerged as a significant predictor for both DME and VH. The optimal cut-off values of IL-8 were established at ≥26.6 pg/mL for DME, and ≥7.96 pg/mL for VH. AH IL-1ra level was identified as a significant predictor for TRD and VH, with cut-off values exceeding 16.1 pg/mL for both complications. In addition, AC flare together with AH IL-4, IL-9, eotaxin, IP-10, and MCP-1 levels were detected as significant predictors for VH. The respective cut-off values of these predictors were 11.1 photons/ms, 0.24 pg/mL, 4.13 pg/mL, 3.55 pg/mL, 306.8 pg/mL, and 264.6 pg/mL or above.

## 3. Discussion

This study examined the complex interrelationship between AC flare, AH cytokines, and VF cytokines in PDR eyes. To assess the intraocular immune status based on inflammatory cytokines, we employed Spearman’s rank correlation analysis. We then utilized multiple regression analysis to identify AH cytokines that causally contribute to elevated AC flare. Finally, we performed ROC curve analysis to evaluate the potential of these cytokines as predictive biomarkers for the complications of DME, TRD, and VH. The study has three key findings. (1) Intraocular IL-8 exhibited consistent positive correlation between AC flare, AH level, and VF level in both PDR and control groups. This finding suggests that IL-8 is a potent universal intraocular biomarker for ocular diseases, which can be derived from AC flare measurement. (2) In the PDR group, multiple regression analysis identified IP-10 and MCP-1 as significant causal cytokines of elevated AC flare. Furthermore, like IL-8, these cytokines also correlated positively between AC flare, AH levels, and VF levels. (3) In PDR eyes, ROC curve analysis identified only AH IL-8 level as a significant predictor of DME. Since AH IL-8 can be inferred from AC flare value, AC flare is potentially a non-invasive biomarker for predicting DME complication.

AH is a transparent, colorless fluid that fills the anterior and posterior chambers of the eye. Secreted by the ciliary body, it flows from the posterior chamber through the pupil into the anterior chamber, where it ultimately drains through the trabecular meshwork at the iridocorneal angle [23]. This continuous flow plays a vital role in maintaining intraocular pressure, supplying nutrients and metabolites to intraocular tissues, and removing waste products [24,25]. The AH composition includes a variety of organic and inorganic ions, carbohydrates, glutathione, urea, amino acids, proteins, dissolved gases (oxygen and carbon dioxide), and water [26]. Previous studies have shown no significant differences in osmolarity, total dissolved solids, or pH between the anterior and posterior chamber fluids [26,27]. The iris acts as a valve, typically preventing backflow from the anterior chamber into the posterior chamber [28]. In recent years, AH that can be sampled in a minimally invasive manner has emerged as a valuable source of biomarkers for investigating the intraocular immune environment in PDR [21] as well as other ocular diseases including AMD [29,30], retinal vein occlusion [31], and retinitis pigmentosa [32].

This study revealed concurrent increases in AC flare and intraocular cytokine levels in PDR eyes compared to controls. Notably, IL-8, eotaxin, IP-10 and MIP-1α levels increased significantly both in the AH and VF of PDR eyes, while IFN-γ, MCP-1, MIP-1β, RANTES and TNFα levels were elevated significantly only in the VF. This disparity in cytokine distribution between the anterior and posterior chambers could be attributed to the distinct flow dynamics of the fluids in the two chambers of the eye. Abnormalities in diabetic capillaries [33] including basement membrane thickening, endothelial cell alterations, and pericyte degeneration contribute to increased vascular permeability [34]. Diabetic retinopathy is a severe manifestation of diabetic microangiopathy [35]. Diabetic ciliopathy and iridopathy are additional complications caused by vascular changes induced by DM [36,37].

Considering the AH flow process [23], AC flare intensity and AH cytokine levels are likely influenced by dysfunctions in both the blood–aqueous barrier (BAB) and the blood–retinal barrier (BRB). Additionally, secretion and metabolism from the iris and other tissues surrounding the AH may play a role. Conversely, VF cytokine levels would be primarily affected by BRB disruption and the activity within the retina. The precise mechanisms underlying the observed disparity between AH and VF cytokine levels remain unclear. Future research investigating the detailed flow dynamics of intraocular fluids and comprehensively analyzing both the secretion and metabolism of intraocular mediators may help to elucidate the differences in composition between anterior and posterior chamber fluids across various ocular diseases.

Intraocular fluids have been a useful source of biomarkers to explore the intraocular immune environment in various ocular diseases including PDR [4,21,38]. The AH offers several advantages over VF for investigating the intraocular immune environment. Compared to VF, AH can be obtained repeatedly and less invasively, making it a more suitable choice for longitudinal studies [39]. Additionally, previous research suggests that BAB disruption precedes BRB dysfunction [40,41]. Earlier BAB disruption in DR strengthens the argument for using AH in analyses. Furthermore, AC flare value was significantly higher in eyes with advanced stages of DR compared to eyes without retinopathy [42] or eyes with background DR [16,43]. Furthermore, increased AC flare intensity is associated with the progression of DR [16,42,43]. In addition, the involvement of low-grade inflammatory responses in DR pathogenesis is well established [44,45]. Consistent with this concept, elevated levels of inflammatory cytokines including AH IL-6 and VEGF have been reported in DR eyes [7,46,47]. Hillier et al. [48] further underscored the potential of AH cytokines as biomarkers by demonstrating a positive correlation between AH intercellular adhesion molecule-1 level and macular volume, and between AH IL-10 level and BCVA in DR eyes with DME. Currently, intravitreal anti-VEGF injection [49], intravitreal triamcinolone acetonide (TA) [50] and sub-Tenon TA [51] represent the standard therapeutic approaches for DME, a sight-threatening complication of DR [52]. In the future, large-scale prospective studies utilizing AC flare and intraocular cytokines would contribute to advancing our understanding of DR pathology in its various stages [53]. These studies may also shed light on the immunological mechanisms underlying the therapeutic effects of anti-VEGF agents [54] and steroid therapy.

A key finding of this study is the positive correlation between AC flare and cytokines not only in AH, but also in VF. While VF cytokines are not directly connected with AC flare, correlation analysis provides a quantitative association between the two variables. A strong correlation allows for mathematical prediction of one variable’s value based on the other [55]. In this study, elevated levels of IL-8, eotaxin, IFN-γ, IP-10, MCP-1, and MIP-1α were observed in the tVF of PDR eyes. Notably, these levels also displayed a positive correlation with AC flare values. These findings are consistent with previous research demonstrating elevated VF levels of inflammatory cytokines including IL-6, IL-8, IP-10, MCP-1, TNFα and VEGF-A in PDR eyes compared with non-diabetic controls [21,38]. Additionally, several studies have suggested associations of the VF levels of IL-6, MCP-1, VEGF, ICAM-1 [56,57] and IL-8 [58] with DME. AC flare, which corresponds to the total protein concentration in the AC, offers a convenient and non-invasive measurement approach [8]. The finding of a correlation between AC flare and VF cytokines in this study suggests the potential for non-invasively estimating intraocular immune status based on AC flare values. This approach may also be valuable in understanding disease pathology and evaluating the efficacy of therapeutic interventions.

Our study also identified several promising biomarkers for predicting the complication risk of DME, TRD and VH in PDR eyes. In particular, ROC curve analysis demonstrated the significant predictive potential of AH IL-8 level for the occurrence of DME. ROC curve analysis also identified AC flare and several AH inflammatory cytokines as potential predictive biomarkers for VH development. AH cytokines offer distinct advantages as intraocular biomarkers. AH can be safely and repeatedly sampled, and cytokine measurements provide valuable longitudinal data [39]. Furthermore, AC flare measurement is a non-invasive procedure, further enhancing its clinical utility [8]. In PDR, the presence of VH can obscure the fundus, making it challenging to detect DME. Early identification of PDR patients at risk of DME and VH is crucial for timely and personalized treatment interventions. By stratifying patients based on their individual risk profiles, ophthalmologists can tailor treatment schedules to optimize patient outcomes and prevent vision loss.

The present study has several limitations. First, the retrospective observational design limits data analysis to medical insurance-covered routine examinations and laboratory tests conducted at the department of ophthalmology. Consequently, potential risk factors for DR such as disease duration of DM, hyperlipidemia, dietary habits, exercise patterns, and smoking history [59] were not included due to incomplete medical records. Additionally, the high prevalence of VH (89.5%) significantly compromised the reliability of ophthalmic findings including BCVA, CRT, and fundus examinations, precluding their use as explanatory variables in multivariate analyses. Second, the relatively small sample size in a single institution limits the generalizability of the findings to the entire population of PDR patients. Third, the low expression levels and detection rates of cytokines such as PDGF-BB, IL-4 and MIP-1α are influenced by a small sample size. As we have imputed values below the detection limit as zero, the calculated values for these cytokines may be underestimated, particularly for those with low detection rates. Therefore, the significance of differences in these cytokines should be interpreted with caution. Fourth, the dynamics of cytokine levels in the eye can vary depending on the timing of sample collection and the specific disease conditions. Therefore, these factors should be considered in future studies. Fifth, the control group should be carefully selected and matched to the study group. While there was no significant difference in the gender ratio between the two groups in our study, a slight male predominance (*p* = 0.192) could potentially affect the results.

A key strength of this study lies in the concurrent examination of AC flare and intraocular cytokines. This approach permitted a comprehensive evaluation of their complex interrelations and influences on PDR pathology through multivariate analyses. Furthermore, the data utilized in this study reflects real-world practices of PDR management, and there was no arbitrary intervention bias in the treatment process.

## 4. Materials and Methods

### 4.1. Subjects

A retrospective observational study was conducted on 19 eyes with PDR in 19 patients with type 2 DM undergoing combined cataract surgery and pars plana vitrectomy (PPV) for VH and/or TRD at the National Defense Medical College between 1 September 2016, and 31 July 2022. PDR was diagnosed according to the international clinical disease severity classification of DR [60]. The control group was composed of 8 eyes (8 patients) with MH, and 11 eyes (11 patients) with ERM.

Inclusion criteria were adopted from previous studies [4,20,38]: (1) no history of intraocular inflammatory and/or ischemic diseases including retinal artery occlusion, retinal vein occlusion, AMD, ocular tumor, uveitis, endophthalmitis, neovascular glaucoma, and dialysis for renal failure; and (2) no prior PPV, ocular trauma, or intravitreal therapies including steroid and anti-VEGF agents. For patients in the PDR or control group who underwent PPV in both eyes, AH and VF specimens collected from the eye that was operated first were used in analyses. The disposition of subjects is summarized in Supplementary Figure S1.

In the PDR group, none of the patients received panretinal photocoagulation (PRP) within 7 days prior to PPV. However, 8 eyes (42.1%) had undergone PRP more than 7 days before PPV. Seventeen eyes (89.5%) presented with VH obscuring fundus details. DME and TRD were observed in 9 eyes (47.4%) and 3 eyes (15.8%), respectively. DME was defined as retinal thickening within the central 3 mm circle on the Early Treatment Diabetic Retinopathy Study (ETDRS) grid [61] in the macula [38], measured by spectral-domain optical coherence tomography (SD-OCT; Cirrus HD-OCT, Carl Zeiss Meditec, Dublin, CA, USA). DME and TRD was confirmed using color fundus photography (CFP), fluorescein angiography (FAG), and SD-OCT before PPV. In patients with dense VH hindering preoperative SD-OCT examination, the procedure was conducted during PPV using an intraoperative OCT system (EnFocus, Leica Microsystems/Bioptigen, Morrisville, NC, USA). All TRD cases involved regions outside the central 6 mm circle of the ETDRS grid in the macula.

An a priori power analysis was conducted based on previous clinical data [38] to determine the sample size required for this study. We calculated effect sizes (Hedges’ g) using VF IL-6 concentration, a representative VF cytokine with a significant difference between PDR eyes and non-DR control eyes (PDR: *n* = 26, IL-6 = 133.2 ± 159.1 pg/mL; ERM or MH control: *n* = 30, IL-6 = 8.53 ± 19.4 pg/mL [38]). The calculated effect sizes (Hedges’ g) for VF IL-6 were 1.14. To detect significant differences in VF IL-6 concentration with a statistical power of 0.80 [62], the required sample size for a two-tailed test is 13.0 [38]. However, for multivariate analyses, the number of samples should be one or more than that of explanatory variables [63]. Therefore, we aimed to recruit at least 15 cases each for the PDR and control groups to allow for potential margins of error.

### 4.2. Diagnostics

Diagnoses of PDR, MH, and ERM were established based on a comprehensive ophthalmic examination including best-corrected visual acuity (BCVA) test using a decimal chart, intraocular pressure measurement, slit-lamp biomicroscopy, dilated fundus examination, and SD-OCT. For PDR patients, CFP and FAG were additionally performed. BCVA was converted to logMAR units for statistical analysis, with counting fingers, hand motion, light perception, and no light perception assigned logMAR values of 1.85, 2.30, 2.80, and 2.90, respectively [64,65]. CRT defined as the mean retinal thickness within the central 1 mm ETDRS grid in the macula [38] was measured by SD-OCT. Three retinal specialists (members of the Japanese Retina and Vitreous Society) independently reviewed clinical findings and confirmed diagnoses. In case of discrepancy among the three assessors, the decision was adjudicated by majority rule.

### 4.3. Measurement of Anterior Chamber Flare

AC flare was measured using an FM-700 laser flare-cell meter (Kowa Company, Ltd., Tokyo, Japan). This instrument emits a 635 nm laser beam into the AC and detects the backscattered light to quantify protein concentration [10]. According to the manufacturer, the meter has a coefficient of variation below 10%, ensuring measurement consistency regardless of the examiner. In this study, we obtained and averaged at least five measurements for each eye.

### 4.4. Intraocular Fluid Collection

Approximately 0.1 mL of undiluted AH was obtained at the beginning of cataract surgery. During PPV, approximately 0.5 mL of undiluted VF was collected using a 25-gauge vitreous cutter inserted into the mid-vitreous cavity prior to active infusion. Collected AH and VF specimens were transferred into separate sterile tubes and stored at −80 °C until analysis. No complications associated with sample collection were observed. Prior to analysis, undiluted VF samples were centrifuged at 10,000× *g* for 10 min. The supernatant (50 μL) was then used in the cytokine assay [4,20,38]. All standards and samples were assayed in duplicate.

### 4.5. Cytokine Measurements

A multiplex bead-based analysis platform (Bio-Plex Suspension Array System; Bio-Rad, Hercules, CA, USA) was employed to measure 27 intraocular cytokines using a multiplex cytokine panel (Bio-Plex Human Cytokine 27-plex panel; Bio-Rad, Hercules, CA, USA). This panel provides comprehensive coverage of inflammatory cytokines comprising PDGF-BB, IL-1β, IL-1ra, IL-2, IL-4, IL-5, IL-6, IL-7, IL-8, IL-9, IL-10, IL-12, IL-13, IL-15, IL-17A, eotaxin, basic fibroblast growth factor, G-CSF, granulocyte macrophage colony-stimulating factor, IFN-γ, IP-10, MCP-1, MIP-1α, MIP-1β, RANTES, TNFα and VEGF-A. The detection limit for each cytokine in the panel is shown in Table 2. Cytokine levels in AH and VF samples below the detectable limit were assigned a value of 0 for statistical analysis [4,20,38].

### 4.6. Statistical Analysis

Statistical analyses were conducted using the statistic add-in software for Excel (BellCurve for Excel^®^, SSRI Co., Ltd., Tokyo, Japan, software version: 4.05, and XLSTAT^®^, Addinsoft Company, Paris, France, software version: Annual version 2021.4.1.1201). Data are presented as mean ± standard deviation [4,20,38]. For comparisons between two unpaired groups, non-parametric tests were employed. The Mann–Whitney U test was used for data comparison, and Spearman’s rank correlation test was used to assess correlation. Pearson’s chi-squared test was used to analyze categorical variables when the expected value in each category was greater than or equal to 4. Cytokines with mean value 0 were excluded as undetectable cytokines in Spearman’s rank correlation test. Additionally, cytokines with detection rates exceeding 20% were included as explanatory variables in multiple regression analysis and ROC curve analysis. The multiple regression analysis utilized a stepwise discriminant analysis with entry and removal criteria set at *p* < 0.05 and *p* < 0.1, respectively [66].

## 5. Conclusions

The present study demonstrates the potential of AC flare and AH cytokines as, respectively, non-invasive and minimally invasive biomarkers for evaluating intraocular immune status and predicting ocular complications in PDR. Intriguingly, both AH and VF IL-8 exhibit significant positive correlation with AC flare in PDR eyes and controls. This finding suggests that IL-8 may serve as a potent and universal intraocular biomarker, potentially quantifiable through non-invasive AC flare assessment. Furthermore, AH IL-8 was detected as a significant predictor of complication risk of both DME and VH. These findings suggest that AH cytokines, particularly IL-8, in conjunction with AC flare may be valuable intraocular biomarkers for elucidating the immunopathology of PDR and predicting sight-threatening complications such as DME. Increased recognition among ophthalmologists regarding the usefulness of the non-invasive AC flare measurement can facilitate closer collaboration with patients, ultimately aiming to prevent the progression of PDR and its vision-threatening complications.

## Figures and Tables

**Table 1:** Demographic and clinical data of eyes with proliferative diabetic retinopathy and controls.

Category	PDR		Control		*p* Value	Unit	Reference Range
*n*	19		19				
	Detectable	Mean ± SD	Detectable	Mean ± SD			
	*n* (%)		*n* (%)				
Age	19 (100)	61.5 ± 10.4	19 (100)	66.1 ± 9.42	0.194	year	
Gender (M/F)	13 (68.4)/6		8 (42.1)/11		0.192		
Laterality (R/L)	8 (42.1)/11		8 (42.1)/11		0.742		
**logMAR VA**	19 (100)	1.87 ± 0.66	19 (100)	0.46 ± 0.33	**5.55 × 10^−5^**		
IOP	19 (100)	13.7 ± 3.72	19 (100)	14.6 ± 2.72	0.202	mmHg	10 to 21
CRT	6 (31.6)	361.7 ± 118.8	19 (100)	458.6 ± 97.6	0.085	μm	
Subgroup							
PRP (+/−)	8 (42.1)/11		–				
Focal PC (+/−)	0 (0)/19		–				
DME (+/−)	9 (47.4)/11		–				
TRD (+/−)	3 (15.8)/16		–				
VH (+/−)	17 (89.5)/2		–				
IVB (+/−)	0 (0)/19		–				
NVG (+/−)	0 (0)/19		–				
MH/ERM	–		8/11				

CRT—central retinal thickness, DME—diabetic macular edema, ERM—idiopathic epiretinal membrane, F—female, IOP—intraocular pressure, IVB—intravitreal injection of bevacizumab, logMAR—logarithm of the minimum angle of resolution, L—left, M—male, MH—idiopathic macular hole, mmHg—millimeter of mercury, *n*—number, NVG—neovascular glaucoma, PC—photocoagulation, PDR—proliferative diabetic retinopathy, PRP—panretinal photocoagulation, R—right, SD—standard deviation, TRD—tractional retinal detachment, VA—visual acuity, VH—vitreous hemorrhage.

**Table 2:** Anterior chamber flare intensity and aqueous humor cytokine levels in eyes with proliferative diabetic retinopathy and controls.

Category	PDR		95% Confidence Interval		Control		95% Confidence Interval		*p* Value	Detection Range		
*n*	19				19							
	Detectable	Mean ± SD	Lower	Upper	Detectable	Mean ± SD	Lower	Upper				
	*n* (%)				*n* (%)							
**AC flare intensity**	19 (100)	21.1 ± 13.9	14.4	27.7	19 (100)	7.08 ± 3.23	5.53	8.64	**4.64 × 10^−5^**	1.00	to	500.0
**Aqueous humor**												
PDGF-BB	nd	0	0	0	nd	0	0	0	–	1.00	to	67,187
IL-1β	nd	0	0	0	nd	0	0	0	–	0.06	to	4598
IL-1ra	14 (73.7)	33.4 ± 40.0	14.1	52.6	15 (78.9)	61.9 ± 110.3	8.77	115.1	0.531	2.52	to	247,147
IL-2	nd	0	0	0	0 (0)	0	0	0	–	0.54	to	26,397
IL-4	4 (21.1)	0.12 ± 0.27	−0.01	0.25	3 (15.8)	0.04 ± 0.10	−0.01	0.09	0.524	0.08	to	4035
IL-5	1 (5.26)	0.94 ± 4.11	−1.04	2.93	2 (10.5)	0.68 ± 2.05	−0.30	1.67	0.536	3.53	to	81,206
IL-6	18 (94.7)	260.6 ± 429.3	53.7	467.5	17 (89.5)	129.6 ± 197.6	34.4	224.9	0.380	0.37	to	21,699
IL-7	7 (36.8)	3.23 ± 5.59	0.54	5.93	10 (52.6)	2.76 ± 3.01	1.31	4.21	0.529	0.49	to	41,077
**IL-8**	19 (100)	31.1 ± 34.6	14.4	47.8	14 (73.7)	5.76 ± 5.27	3.22	8.30	**0.003**	0.75	to	27,477
IL-9	4 (21.1)	3.44 ± 7.71	−0.27	7.16	1 (5.26)	0.24 ± 1.06	−0.27	0.75	0.395	0.92	to	45,633
IL-10	nd	0	0	0	nd	0	0	0	–	0.74	to	27,194
IL-12	nd	0	0	0	nd	0	0	0	–	1.17	to	21,022
IL-13	1 (5.26)	0.08 ± 0.35	−0.09	0.25	nd	0	0	0	0.534	0.29	to	9203
IL-15	nd	0	0	0	1 (5.26)	0.54 ± 2.34	−0.59	1.67	0.534	1.62	to	251,038
IL-17A	1 (5.26)	0.56 ± 2.46	−0.62	1.75	nd	0	0	0	0.534	1.69	to	41,194
**Eotaxin**	19 (100)	7.41 ± 5.17	4.92	9.90	18 (94.7)	2.73 ± 1.93	1.80	3.67	**0.003**	0.07	to	7488
bFGF	nd	0	0	0	nd	0	0	0	–	3.02	to	63,088
G-CSF	3 (15.8)	8.63 ± 29.5	−5.57	22.8	2 (10.5)	3.35 ± 12.0	−2.44	9.15	0.534	1.67	to	13,8193
GM-CSF	nd	0	0	0	nd	0	0	0	–	0.38	to	10,559
IFN-γ	18 (94.7)	15.0 ± 15.7	7.44	22.5	17 (89.5)	8.26 ± 9.73	3.57	13.0	0.076	0.35	to	20,941
**IP-10**	19 (100)	1108.7 ± 1664.5	306.5	1911.0	18 (94.7)	183.4 ± 226.4	74.3	292.5	**0.012**	2.75	to	48,834
MCP-1	19 (100)	479.4 ± 314.3	327.9	630.9	19 (100)	404.3 ± 324.5	247.9	560.7	0.294	0.44	to	11,213
**MIP-1α**	14 (73.7)	0.45 ± 0.38	0.26	0.63	9 (47.4)	0.19 ± 0.25	0.07	0.31	**0.0495**	0.04	to	1045
MIP-1β	19 (100)	8.60 ± 4.69	6.34	10.9	19 (100)	6.62 ± 3.77	4.80	8.44	0.242	0.29	to	8095
RANTES	4 (21.1)	6.00 ± 15.5	−1.46	13.5	nd	0	0	0	0.277	1.41	to	51,984
TNFα	3 (15.8)	3.46 ± 9.54	−1.14	8.06	1 (5.26)	0.69 ± 2.99	−0.76	2.13	0.509	2.73	to	63,996
VEGF-A	4 (21.1)	19.0 ± 40.1	−0.37	38.3	3 (15.8)	12.3 ± 36.2	−5.09	29.8	0.531	2.42	to	209,528

AC flare intensity is expressed in photon counts per millisecond (ph/ms). Cytokine levels are expressed in pg/mL. AC—anterior chamber, bFGF—basic fibroblast growth factor, G-CSF—granulocyte colony-stimulating factor, GM-CSF—granulocyte macrophage colony-stimulating factor, IFN—interferon, IP-10—interferon gamma-induced protein 10, IL—interleukin, MIP—macrophage inflammatory protein, MCP—monocyte chemotactic protein, nd—not detected, PDGF—platelet derived growth factor, ra—receptor antagonist, RANTES—regulated on activation, normal T-cell expressed and secreted, TNF—tumor necrosis factor, VEGF—vascular endothelial growth factor.

**Table 3:** Anterior chamber flare intensity and vitreous fluid cytokine levels in eyes with proliferative diabetic retinopathy and controls.

Category	PDR		95% Confidence Interval		Control		95% Confidence Interval		*p* Value	Detection Range		
*n*	19				19							
	Detectable	Mean ± SD	Lower	Upper	Detectable	Mean ± SD	Lower	Upper				
	Rate (%)				Rate (%)							
**AC Flare**	19 (100)	21.1 ± 13.9	14.4	27.7	19 (100)	7.08 ± 3.23	5.53	8.64	**4.64 × 10^−5^**	1.00	to	500.0
**Vitreous fluid**												
PDGF-BB	5 (26.3)	15.2 ± 43.4	−5.71	36.1	nd	0	0	0	0.178	1.00	to	67,187
IL-1β	3 (15.8)	0.07 ± 0.19	−0.02	0.17	nd	0	0	0	0.411	0.06	to	4598
IL-1ra	16 (84.2)	28.6 ± 20.6	18.7	38.5	15 (78.9)	16.2 ± 14.0	9.43	22.9	0.060	2.52	to	247,147
IL-2	1 (5.26)	0.10 ± 0.43	−0.11	0.31	nd	0	0	0	0.534	0.54	to	26,397
IL-4	6 (31.6)	0.20 ± 0.37	0.03	0.38	4 (21.1)	0.02 ± 0.05	≈0	0.05	0.373	0.08	to	4035
IL-5	2 (10.5)	1.33 ± 4.57	−0.87	3.54	nd	0	0	0	0.511	3.53	to	81,206
**IL-6**	13 (68.4)	57.5 ± 106.0	6.44	108.7	8 (42.1)	7.81 ± 24.9	−4.17	19.8	**0.041**	0.37	to	21,699
IL-7	17 (89.5)	18.3 ± 10.5	13.2	23.3	18 (94.7)	15.1 ± 9.34	10.6	19.6	0.271	0.49	to	41,077
**IL-8**	19 (100)	102.3 ± 133.2	38.1	166.5	14 (73.7)	3.87 ± 3.65	2.11	5.63	**1.25 × 10^−5^**	0.75	to	27,477
IL-9	6 (31.6)	3.42 ± 6.50	0.28	6.55	nd	0	0	0	0.106	0.92	to	45,633
IL-10	2 (10.5)	0.25 ± 0.85	−0.15	0.66	nd	0	0	0	0.511	0.74	to	27,194
IL-12	nd	0	0	0	nd	0	0	0	–	1.17	to	21,022
IL-13	4 (21.1)	0.71 ± 1.82	−0.17	1.59	4 (21.1)	0.35 ± 0.72	0.01	0.70	0.545	0.29	to	9203
IL-15	nd	0	0	0	nd	0	0	0	–	1.62	to	251,038
IL-17A	3 (15.8)	0.91 ± 2.19	−0.14	1.97	nd	0	0	0	0.411	1.69	to	41,194
**Eotaxin**	19 (100)	12.1 ± 8.01	8.23	16.0	19 (100)	3.88 ± 1.72	3.05	4.71	**3.40 × 10^−5^**	0.07	to	7488
bFGF	1 (5.26)	0.25 ± 1.07	−0.27	0.76	nd	0	0	0	0.534	3.02	to	63,088
G-CSF	5 (26.3)	15.5 ± 37.0	−2.34	33.3	3 (15.8)	1.70 ± 4.08	−0.27	3.67	0.463	1.67	to	138,193
GM-CSF	1 (5.26)	0.06 ± 0.25	−0.06	0.18	nd	0	0	0	0.534	0.38	to	10,559
**IFN-γ**	19 (100)	34.3 ± 32.4	18.7	49.9	15 (78.9)	4.35 ± 3.90	2.47	6.23	**1.74 × 10^−4^**	0.35	to	20,941
**IP-10**	19 (100)	3033.2 ± 5035.2	606.4	5460.1	18 (94.7)	467.3 ± 378.0	285.1	649.5	**7.56 × 10^−4^**	2.75	to	48,834
**MCP-1**	19 (100)	789.2 ± 605.8	497.2	1081.2	19 (100)	247.9 ± 82.0	208.4	287.4	**1.93 × 10^−4^**	0.44	to	11,213
**MIP-1α**	18 (94.7)	1.21 ± 1.29	0.58	1.83	5 (26.3)	0.06 ± 0.11	0.01	0.11	**2.62 × 10^−5^**	0.04	to	1045
**MIP-1β**	18 (94.7)	8.33 ± 7.21	4.86	11.8	19 (100)	2.61 ± 2.13	1.59	3.64	**0.004**	0.29	to	8095
**RANTES**	10 (52.6)	11.9 ± 20.4	2.07	21.7	nd	0	0	0	**0.009**	1.41	to	51,984
**TNFα**	9 (47.4)	19.5 ± 39.3	0.58	38.4	2 (10.5)	1.73 ± 5.61	−0.97	4.44	**0.0495**	2.73	to	63,996
VEGF-A	6 (31.6)	228.6 ± 661.5	−90.3	547.4	1 (5.26)	0.26 ± 1.12	−0.28	0.80	0.155	2.42	to	209,528

**Table 4:** Correlation matrices of significance levels obtained by Spearman’s rank correlation analyses of anterior chamber flare intensity and aqueous humor cytokine levels in eyes with proliferative diabetic retinopathy and controls.

**PDR**																					
	**AC Flare**	**IL-1ra**	**IL-4**	**IL-5**	**IL-6**	**IL-7**	**IL-8**	**IL-9**	**IL-13**	**IL-17A**	**Eotaxin**	**G-CSF**	**IFN-γ**	**IP-10**	**MCP-1**	**MIP-1α**	**MIP-1β**	**RANTES**	**TNFα**	**VEGF-A**	
**AC Flare**	-	0.326	0.459	0.286	0.403	0.786	**0.032**	0.560	0.376	0.376	**0.007**	0.814	**0.029**	**0.001**	**0.047**	0.466	0.297	0.143	**0.022**	0.920	
IL-1ra		-	0.769	0.145	0.735	0.513	0.553	0.094	N/A	N/A	0.175	0.181	0.243	0.139	0.374	0.148	0.292	0.245	0.807	0.392	
**IL-4**			-	**0.016**	**0.004**	0.110	0.295	0.065	0.622	0.622	0.357	0.862	0.516	0.325	0.968	0.667	0.212	0.135	0.708	0.295	
**IL-5**				-	0.101	0.476	0.149	**0.016**	0.821	0.821	0.286	0.679	0.598	0.376	0.861	0.595	0.481	0.072	0.679	0.623	
**IL-6**					-	**0.015**	0.904	0.123	0.101	0.101	0.283	0.888	0.304	0.185	0.098	0.444	**0.008**	0.425	0.814	0.437	
**IL-7**						-	0.795	0.449	0.055	0.055	**0.016**	0.184	0.707	**0.027**	0.415	0.429	0.112	0.111	0.216	0.111	
**IL-8**							-	0.056	0.861	0.861	0.099	0.814	**0.002**	0.154	**3.66 × 10^−5^**	**0.004**	0.260	**0.013**	0.283	0.952	
**IL-9**								-	0.623	0.623	0.602	0.060	0.952	0.602	0.637	0.550	**0.008**	**1.34 × 10^−4^**	0.364	0.955	
**IL-13**									-	N/A^#^	0.598	0.679	0.481	0.726	0.598	0.206	0.101	0.623	**0.006**	0.623	
**IL-17A**										-	0.598	0.679	0.481	0.726	0.598	0.206	0.101	0.623	**0.006**	0.623	
**Eotaxin**											-	0.166	0.149	**1.86 × 10^−8^**	0.077	0.219	0.132	0.520	0.911	0.193	
**G-CSF**												-	0.665	0.297	0.973	0.120	0.078	**0.025**	0.446	**0.033**	
**IFN-γ**													-	0.119	**3.89 × 10^−6^**	**0.009**	0.141	0.408	0.076	0.637	
**IP-10**														-	0.067	0.294	0.152	0.659	0.516	0.368	
**MCP-1**															-	**0.005**	0.065	0.085	0.435	0.764	
**MIP-1α**																-	**5.88 × 10^−4^**	0.770	0.494	0.427	
**MIP-1β**																	-	0.083	0.612	0.652	
**RANTES**																		-	0.364	0.229	
**TNFα**																			-	0.759	
**VEGF-A**																				-	
**Control**																					
	**AC Flare**	**IL-1ra**	**IL-4**	**IL-5**	**IL-6**	**IL-7**	**IL-8**	**IL-9**	**IL-15**	**Eotaxin**	**G-CSF**	**IFN-γ**	**IP-10**	**MCP-1**	**MIP-1α**	**MIP-1β**	**TNFα**	**VEGF-A**			
**AC Flare**	-	0.443	0.727	0.658	0.052	0.419	**0.028**	0.598	0.598	0.342	0.658	**0.014**	0.847	**0.006**	0.195	**0.014**	0.101	0.727			
IL-1ra		-	0.225	0.706	0.341	0.182	0.281	0.208	0.860	0.563	0.706	0.353	0.409	0.298	0.949	0.698	0.479	0.225			**0.75** **~** **1**
**IL-4**			-	**2.85 × 10^−4^**	**0.014**	0.383	0.169	0.679	**0.017**	0.596	**2.85 × 10^−4^**	0.064	0.524	0.078	**0.028**	0.275	0.679	N/A^#^			**0.50~0.75**
**IL-5**				-	0.085	**0.047**	0.429	0.743	**4.27 × 10^−4^**	0.820	N/A^#^	**0.042**	0.925	0.080	0.137	0.872	0.743	**2.85 × 10^−4^**			**0.25~0.50**
**IL-6**					-	0.510	**0.011**	0.101	0.285	0.145	0.085	**0.003**	0.134	**1.91 × 10^−4^**	**0.002**	**9.10 × 10^−5^**	N/A	**0.014**			**0~0.25**
**IL-7**						-	0.125	0.348	0.257	0.457	**0.047**	0.274	0.506	0.838	0.722	0.474	0.183	0.383			**−0.25~0**
**IL-8**							-	0.098	0.477	**0.046**	0.429	**0.003**	**0.032**	**0.006**	**0.012**	**0.015**	0.595	0.169			**−0.50~−0.25**
IL-9								-	0.821	0.598	0.743	0.101	0.598	0.101	0.503	0.101	0.821	0.679			**−0.75~−0.50**
**IL-15**									-	0.861	**4.27 × 10^−4^**	0.149	0.726	0.149	0.073	0.598	0.821	**0.017**			**−1~−0.75**
**Eotaxin**										-	0.820	0.899	**1.73 × 10^−7^**	0.399	0.142	**0.039**	0.210	0.596			
**G-CSF**											-	**0.042**	0.925	0.080	0.137	0.872	0.743	**2.85 × 10^−4^**			
**IFN-γ**												-	0.819	**4.30 × 10^−6^**	**0.016**	**0.036**	0.481	0.064			
**IP-10**													-	0.753	0.142	0.055	0.376	0.524			
**MCP-1**														-	**0.007**	**0.003**	0.726	0.078			
**MIP-1α**															-	**4.50 × 10^−4^**	0.245	**0.028**			
**MIP-1β**																-	0.210	0.275			
TNFα																	-	0.679			
**VEGF-A**																		-			

Numerical data are *p* values computed by Spearman’s rank correlation test. Combinations exhibiting significant correlations are highlighted using a color scale. Color scale bar indicates values of Spearman’s rank correlation coefficients. Color scale: low values, dark red; middle to high values, transitioning from pink and light blue to dark blue. N/A—not applicable because correlation coefficient is lower than 1.0 × 10^−10^, N/A^#^—not applicable because absolute value of correlation coefficient is close to 1.

**Table 5:** Associations of anterior chamber flare intensity with aqueous humor cytokine levels by multiple regression analysis in eyes with proliferative diabetic retinopathy and controls.

**PDR**							
**R**	**Corrected R**	**R^2^**	**Corrected R^2^**	**Durbin–Watson Ratio**		**AIC**	***p* Value**
0.767	0.712	0.589	0.506	2.366		90.00	**0.003**
**AC Flare**	**PRC**	**SE**	**Standard PRC**	**95% Confidence Interval**		***t* Value**	***p* Value**
				**Lower**	**Upper**		
IL-7	0.843	0.444	0.340	−0.104	1.790	1.896	0.077
**IP-10**	0.005	0.001	0.541	0.001	0.008	3.090	**0.007**
**MCP-1**	0.024	0.008	0.551	0.008	0.040	3.208	**0.006**
**Control**							
**R**	**Corrected R**	**R^2^**	**Corrected R^2^**	**Durbin–Watson Ratio**		**AIC**	***p* Value**
0.634	0.572	0.402	0.327	2.524		39.80	**0.016**
**AC Flare**	**PRC**	**SE**	**Standard PRC**	**95% Confidence Interval**		***t* Value**	***p* Value**
				**Lower**	**Upper**		
**IL-7**	0.468	0.214	0.436	0.016	0.921	2.193	**0.043**
**MIP-1β**	0.493	0.171	0.574	0.131	0.855	2.887	**0.011**

AIC—Akaike’s information criterion, PRC—partial regression coefficient, R—multiple correlation coefficient, R^2^—coefficient of determination, SE—standard error.

**Table 6:** Correlation matrices of significance levels obtained by Spearman’s rank correlation analyses of anterior chamber flare intensity and vitreous fluid cytokine levels in eyes with proliferative diabetic retinopathy and controls.

**PDR**																										
	**AC Flare**	**PDGF-BB**	**IL-1β**	**IL-1ra**	**IL-2**	**IL-4**	**IL-5**	**IL-6**	**IL-7**	**IL-8**	**IL-9**	**IL-10**	**IL-13**	**IL-17A**	**Eotaxin**	**bFGF**	**G-CSF**	**GM-CSF**	**IFN-γ**	**IP-10**	**MCP-1**	**MIP-1α**	**MIP-1β**	**RANTES**	**TNFα**	**VEGF-A**
**AC Flare**	–	**0.019**	0.052	**0.030**	0.286	**0.039**	0.289	0.169	0.509	**0.047**	0.387	0.086	0.224	0.299	**0.047**	0.286	0.971	0.101	**0.016**	**0.024**	**0.003**	**0.021**	0.154	0.415	**0.014**	0.585
**PDGF-BB**		–	**0.019**	0.053	**0.029**	**6.11 × 10^−4^**	**0.010**	0.162	0.156	**0.024**	0.151	**0.001**	0.371	0.700	**0.028**	**0.029**	0.265	0.571	**0.039**	**0.027**	0.051	**0.015**	**0.019**	0.153	0.088	**5.26 × 10^−4^**
**IL-1β**			–	0.434	**0.006**	**4.13 × 10^−4^**	0.093	**0.003**	0.116	0.835	0.177	**7.06 × 10^−6^**	0.553	0.406	**0.005**	**0.006**	0.470	0.679	0.217	**0.004**	0.431	0.185	0.079	**0.022**	0.214	**0.035**
**IL-1ra**				–	0.209	0.578	0.563	0.577	0.483	**0.004**	0.215	0.143	0.114	**0.043**	0.853	0.209	0.879	0.598	**0.011**	0.518	**0.001**	**0.018**	0.066	0.859	**0.013**	0.608
**IL-2**					–	**0.042**	**4.27 × 10^−4^**	0.203	0.101	0.598	0.075	**4.27 × 10^−4^**	0.072	**0.040**	0.101	N/A^#^	**0.029**	0.821	0.286	0.210	0.481	0.598	0.149	0.082	0.247	**0.043**
**IL-4**						–	**0.013**	**0.005**	0.348	0.289	0.060	**0.003**	0.448	0.822	**2.63 × 10^−4^**	**0.042**	0.112	0.522	0.097	**0.007**	0.293	0.096	0.111	**0.032**	0.279	**0.004**
**IL-5**							–	0.275	**0.040**	0.601	**0.018**	**0.029**	**0.010**	0.223	0.104	**4.27 × 10^−4^**	**0.001**	0.743	0.454	0.229	0.591	0.601	0.107	**0.029**	0.778	**0.006**
**IL-6**								–	0.908	0.546	0.686	**0.028**	0.503	0.417	**0.020**	0.203	0.137	0.238	0.755	**0.042**	0.839	0.498	**0.019**	**0.022**	0.950	**0.022**
**IL-7**									–	0.833	0.842	0.329	0.462	0.766	0.369	0.101	0.503	0.286	0.889	0.115	0.889	0.657	0.389	0.549	0.781	0.665
**IL-8**										–	0.098	0.372	0.120	0.114	0.562	0.598	0.369	0.376	**1.10 × 10^−4^**	0.221	**9.60 × 10^−5^**	**3.19 × 10^−5^**	0.087	0.748	**0.002**	0.481
**IL-9**											–	**0.046**	**3.01 × 10^−6^**	**1.40 × 10^−4^**	0.202	0.075	0.109	0.124	0.129	0.275	0.244	0.117	0.143	**0.001**	0.052	**0.024**
**IL-10**												–	0.323	0.223	**0.019**	**4.27 × 10^−4^**	0.234	0.743	0.289	**0.030**	0.434	0.203	0.059	**0.012**	0.197	**0.003**
**IL-13**													–	**3.47 × 10^−7^**	0.520	0.072	0.223	**0.016**	0.081	0.419	0.089	0.186	0.305	**0.020**	**0.021**	0.364
**IL-17A**														–	0.681	**0.040**	0.700	**0.010**	0.056	0.501	0.069	0.183	0.441	0.067	**0.002**	0.822
**Eotaxin**															–	0.101	0.342	0.861	0.113	**9.23 × 10^−4^**	0.209	0.110	0.085	**0.004**	0.067	**0.040**
**bFGF**																–	**0.029**	0.821	0.286	0.210	0.481	0.598	0.149	0.082	0.247	**0.043**
**G-CSF**																	–	0.571	0.290	0.172	0.573	0.280	**0.023**	0.181	0.978	**0.047**
**GM-CSF**																		–	0.210	0.861	0.210	0.481	0.598	0.515	0.074	0.522
**IFN-γ**																			–	**0.002**	**8.39 × 10^−10^**	**1.61 × 10^−7^**	**0.016**	0.583	**2.30 × 10^−5^**	0.603
**IP-10**																				–	**0.005**	**6.81 × 10^−4^**	**9.23 × 10^−4^**	**0.020**	**0.006**	0.271
**MCP-1**																					–	**3.50 × 10^−7^**	**0.009**	0.694	**1.15 × 10^−5^**	0.911
**MIP-1α**																						–	**3.71 × 10^−4^**	0.435	**6.55 × 10^−5^**	0.322
**MIP-1β**																							–	**0.034**	**0.016**	**0.042**
**RANTES**																								–	0.214	**0.006**
**TNFα**																									–	0.852
**VEGF-A**																										–
**Control**																										
	**AC Flare**	**IL-1ra**	**IL-4**	**IL-6**	**IL-7**	**IL-8**	**IL-13**	**Eotaxin**	**G-CSF**	**IFN-γ**	**IP-10**	**MCP-1**	**MIP-1α**	**MIP-1β**	**TNFα**	**VEGF-A**										
**AC Flare**	–	0.191	0.900	0.830	**0.002**	**0.038**	0.944	**0.013**	0.491	0.388	0.318	0.379	0.389	0.273	**0.019**	0.210										
**IL-1ra**		–	0.500	0.616	0.492	0.757	0.365	0.501	0.879	0.762	**0.008**	0.194	0.333	0.476	0.767	0.373			**0.75~1**							
**IL-4**			–	**2.60 × 10^−4^**	0.344	0.229	0.278	0.678	**1.04 × 10^−4^**	**0.035**	0.189	0.381	**1.74 × 10^−7^**	0.226	0.472	**0.036**			**0.50~0.75**							
**IL-6**				–	0.654	0.293	0.315	0.699	**0.007**	0.332	0.275	0.697	**1.24 × 10^−4^**	0.437	0.941	0.065			**0.25~0.50**							
**IL-7**					–	0.173	0.880	0.311	0.761	0.853	0.080	0.714	0.846	0.310	0.287	0.147			**0~0.25**							
**IL-8**						–	0.976	0.061	0.923	0.100	0.422	0.078	0.572	**0.006**	0.117	0.281			**−0.25~0**							
**IL-13**							–	0.266	0.364	0.900	0.567	0.704	0.722	0.254	0.427	0.623			**−0.50~−0.25**							
**Eotaxin**								–	0.124	0.497	**0.005**	0.590	0.622	0.116	0.142	0.123			**−0.75~−0.50**							
**G-CSF**									–	**0.005**	0.577	0.143	**1.86 × 10^−5^**	0.727	0.546	**0.017**			**−1~−0.75**							
**IFN-γ**										–	0.097	**4.45 × 10^−6^**	**0.028**	**0.031**	0.340	0.147										
**IP-10**											–	0.533	0.194	0.139	0.648	0.101										
**MCP-1**												–	0.520	0.163	0.894	0.286										
**MIP-1α**													–	0.435	0.407	**0.041**										
**MIP-1β**														–	0.135	0.376										
**TNFα**															–	0.743										
**VEGF-A**																–										

**Table 7:** Parameter values obtained from Spearman’s rank correlation analysis of aqueous humor and vitreous fluid cytokine levels in eyes with proliferative diabetic retinopathy and controls.

PDR						Control					
	r	R^2^	95% Confidence Interval		*p* Value		r	R^2^	95% Confidence Interval		*p* Value
			Lower	Upper					Lower	Upper	
IL-1ra	0.231	0.053	−0.256	0.624	0.341	**IL-1ra**	0.553	0.305	0.096	0.817	**0.016**
**IL-4**	0.515	0.265	0.048	0.797	**0.026**	IL-4	0.059	0.003	−0.406	0.500	0.811
IL-5	−0.081	0.007	−0.517	0.388	0.743						
IL-6	0.353	0.124	−0.136	0.703	0.139	IL-6	0.348	0.121	−0.141	0.700	0.145
IL-7	0.051	0.003	−0.413	0.494	0.838	IL-7	−0.223	0.050	−0.618	0.263	0.358
**IL-8**	0.847	0.718	0.589	0.949	**4.68 × 10^−6^**	**IL-8**	0.584	0.342	0.138	0.833	**0.010**
IL-9	−0.072	0.005	−0.510	0.396	0.771						
IL-17A	−0.102	0.010	−0.532	0.371	0.678						
**Eotaxin**	0.654	0.428	0.238	0.867	**0.003**	**Eotaxin**	0.573	0.329	0.123	0.828	**0.012**
**G-CSF**	0.707	0.500	0.322	0.892	**0.001**	G-CSF	0.342	0.117	−0.147	0.696	0.152
**IFN-γ**	0.719	0.517	0.341	0.897	**0.001**	IFN-γ	0.250	0.062	−0.238	0.637	0.301
**IP-10**	0.919	0.845	0.762	0.974	**1.15 × 10^−6^**	IP-10	0.142	0.020	−0.336	0.562	0.560
**MCP-1**	0.830	0.689	0.551	0.942	**3.22 × 10^−6^**	MCP-1	0.135	0.018	−0.342	0.557	0.580
**MIP-1α**	0.614	0.378	0.180	0.848	**0.006**	MIP-1α	−0.230	0.053	−0.623	0.257	0.343
MIP-1β	0.274	0.075	−0.215	0.653	0.256	MIP-1β	0.226	0.051	−0.260	0.621	0.350
RANTES	0.315	0.099	−0.174	0.679	0.189						
TNFα	0.267	0.071	−0.222	0.648	0.269	**TNFα**	0.726	0.528	0.354	0.900	**0.001**
**VEGF-A**	0.489	0.239	0.016	0.783	**0.035**	VEGF-A	−0.102	0.010	−0.532	0.371	0.678

r—Spearman’s rank correlation coefficient, R^2^—coefficient of determination.

**Table 8:** Parameter values of significant biomarkers in receiver operating characteristic analysis for occurrence of diabetic macular edema, traction retinal detachment and vitreous hemorrhage.

DME	AUC	SE	95% Confidence Interval		Chi-Square Value	*p* Value	Cut-Off Value	TPF	FPF	Specificity	OR	PPV	NPV
			Lower	Upper									
IL-8	**0.74**	0.12	0.51	0.98	4.22	**0.040**	**26.6**	0.67	0.30	0.70	4.67	66.7	70.0
**TRD**													
IL-1ra	**0.74**	0.12	0.51	0.97	4.19	**0.041**	**16.1**	1.00	0.38	0.63	–	33.6	100
**VH**													
AC flare	**0.94**	0.06	0.83	1.06	56.2	**6.38 × 10^−14^**	**11.1**	0.94	0	1.00	–	100	66.5
IL-1ra	**0.79**	0.12	0.56	1.03	6.04	**0.014**	**16.1**	0.71	0	1.00	–	100	28.5
IL-4	**0.62**	0.05	0.51	0.72	4.92	**0.027**	**0.24**	0.24	0	1.00	–	100	13.3
IL-8	**0.82**	0.10	0.62	1.03	9.78	**0.002**	**7.96**	0.76	0	1.00	–	100	33.3
IL-9	**0.62**	0.05	0.51	0.72	4.92	**0.027**	**4.13**	0.24	0	1.00	–	100	13.3
Eotaxin	**0.91**	0.10	0.72	1.11	16.9	**4.02 × 10^−5^**	**3.55**	0.82	0	1.00	–	100	40.0
IP-10	**0.82**	0.10	0.62	1.03	9.78	**0.002**	**306.8**	0.76	0	1.00	–	100	33.3
MCP-1	**0.88**	0.13	0.63	1.14	8.78	**0.003**	**264.6**	0.76	0	1.00	–	100	33.3

All cytokines in this table are aqueous humor cytokines. The cut-off point was set to the closest point to the upper left-hand corner of the graph. AUC—area under the curve, FPF—false positive fraction, NPV—negative predictive value, OR—odds ratio, PPV—positive predictive value, SE—standard error. TPF—true positive fraction.

## Data Availability

The data sets used and analyzed during the current study are available from the corresponding author upon reasonable request.

## Supplementary Materials

The following supporting information can be downloaded at: https://www.mdpi.com/article/10.3390/ijms25179158/s1.
