# Supplementary material for: Anterior Chamber Flare as a Non-Invasive Assessment of Intraocular Immune Status and Ocular Complications in Proliferative Diabetic Retinopathy
Source: Int J Mol Sci. 2024 Aug 23;25(17):9158. doi: 10.3390/ijms25179158 (PMC11394674; doi:10.3390/ijms25179158)
Supplement: Supplementary file 1 [file ijms-25-09158-s001.zip › ijms-3126052-supplementary.pdf]

Supplementary Materials

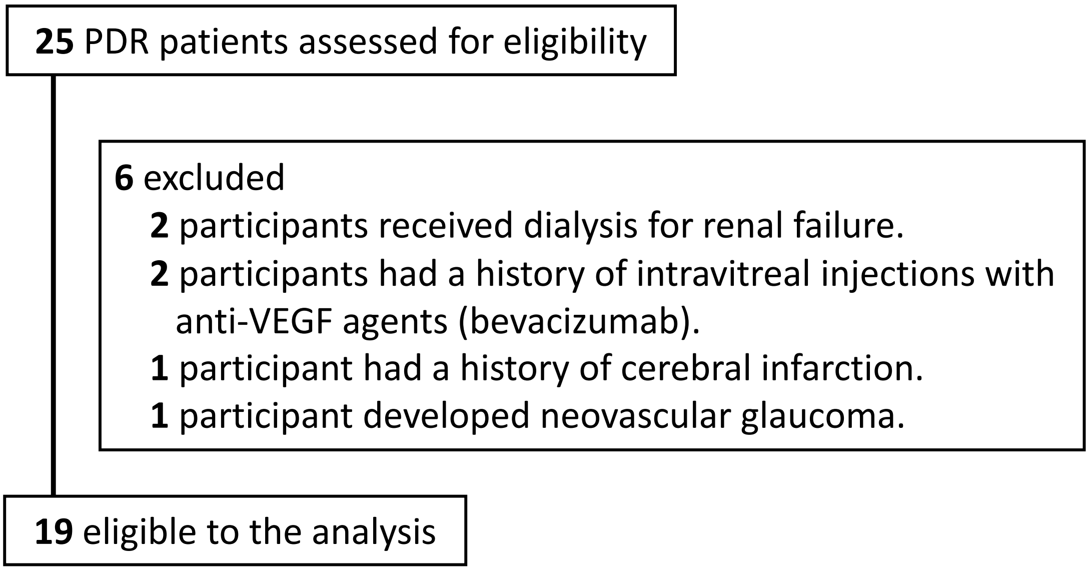

Figure S1. Trial profile. Flowchart of patients with proliferative diabetic retinopathy enrolled for pars plana vitrectomy is shown. Finally, 19 patients were eligible for analysis in this study. PDR; proliferative diabetic retinopathy, VEGF; vascular endothelial growth factor.
